# SARS-CoV-2 and Portal Vein Thrombosis: A Rare Gastrointestinal Manifestation of COVID-19

**DOI:** 10.7759/cureus.14340

**Published:** 2021-04-07

**Authors:** Sindhura Kolli, Veeral M Oza

**Affiliations:** 1 Internal Medicine, New York University Langone Hospital, New York, USA; 2 Gastroenterology, University of South Carolina-Greenville, Greenville, USA

**Keywords:** covid-19, portal vein thrombosis, pvt, sars-cov-2, gastroenterology

## Abstract

Portal vein thrombosis is defined as a clot within the trunk or intrahepatic branches of the portal vein. Sequelae involves either partial or complete recanalization. However, in patients with liver disease, it can progress to a cavernoma instead of recanalization. This can result in gastrointestinal bleeding and intestinal infarction. Its rising incidence in severe acute respiratory syndrome coronavirus 2 is an important clinical aspect that needs to be addressed and treated.

## Introduction

Coagulopathy in severe acute respiratory syndrome coronavirus 2 (SARS-CoV-2) or coronavirus disease 2019 (COVID-19) was a disturbing discovery as the incidence of micro- and macrovasculature thrombi increased in proportion to the rising number of SARS-CoV-2 cases. While accounts of deep vein thrombosis (DVT) and pulmonary emboli were regularly added to the COVID-19 literature, only a handful incidences of portal vein thrombosis (PVT) have been reported thus far [1]. This rare but prevalent gastrointestinal effect of COVID-19 should be recognized as a possible etiology of abdominal pain for early recognition and treatment to prevent complications and further sequelae.

## Case presentation

**Figure 1:**
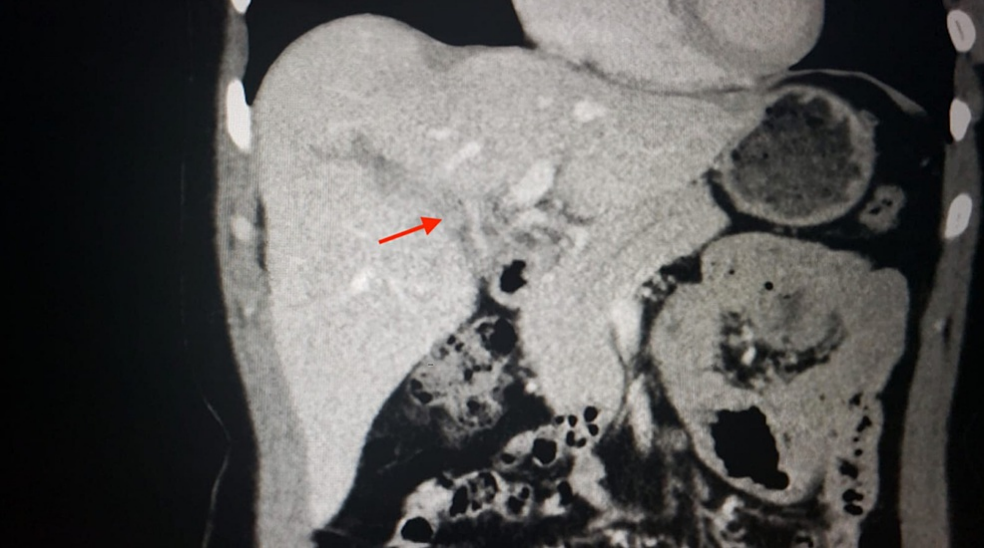
CT imaging of PVT in a COVID-19 positive patient. CT, computed tomography; PVT, portal vein thrombosis; COVID-19, coronavirus disease 2019

A 44-year-old female patient with no past medical history presented to the emergency department (ED) with abdominal pain, abdominal bloating, and chest discomfort. Abdominal pain in the epigastric and right upper quadrants was dull, aching, and was present for one to two weeks prior to admission. It was accompanied by bloating and no alleviating factors. She visited her primary care physician, who performed a right upper quadrant ultrasound that was concerning for a PVT and sent her to the hospital. She denied any calf pain, recent surgery, immobilization, or any other risk factors for a possible DVT. PVT was confirmed in the ED using a computed tomography (CT) (Figure 1).

Her vitals were within normal limits and stable. Her labs, including her hepatic workup, were also unremarkable. She was placed on a therapeutic heparin drip and coumadin. Hypercoagulable workup outside of prothrombin time, activated partial thromboplastin time, fibrinogen, and platelet count included lupus anticoagulant panels; activated protein C resistance, protein C, and protein S activity; and antithrombin activity were negative. Specific coagulation factor levels were not indicated as preliminary workup was negative. CT of her abdomen did not demonstrate any other provoking factors, including cirrhosis, for a thrombosis. She was subsequently discharged on coumadin for economic reasons. Her COVID-19 test performed during her admission returned positive after she was discharged. Upon discharge, she also developed a dry cough as a late presentation of her COVID-19. She was counseled on a vitamin K-appropriate diet as well as common medication interactions with coumadin and scheduled for outpatient follow-up post appropriate quarantine.

## Discussion

The commonly reported gastrointestinal manifestations of COVID-19 include nausea, vomiting, diarrhea, abdominal pain, and to a lesser extent abnormal liver function tests (LFTs). Abnormal LFTs could be secondary to sepsis-related inflammation or direct viral-associated hepatocellular assault through entry of the SARS-CoV-2 virus via ACE-2 receptors expressed on hepatocytes and hepatic cholangiocytes. Similarly, the inflammatory state incitation of a cytokine storm, complement activation, and coagulation cascade is thought to create a pro-coagulopathic state, leading to a higher incidence of venous thrombosis and, less commonly, arterial thrombosis. Both gastrointestinal manifestations and the presence of venous or arterial thromboembolism bear poorer outcomes in COVID-19 patients [2,3].

PVT is not a surprising sequelae given it often occurs against the backdrop of an inflammatory state, such as cirrhosis, or when a prothrombotic predisposing condition is identified. PVT presents with nonspecific symptoms such as abdominal pain, fever, small-volume abdominal ascites, and splenomegaly [4]. However, when complications such as intestinal ischemia, bowel infarction, and ileus arise, the mortality rate plummets from an 85% five-year survival rate to 20-50% rapidly [5,6]. Labs demonstrate normal LFTs, except for an elevated alkaline phosphatase if biliary pathology occurs, with prolonged prothrombin time, decreased coagulation factors, and an increase in D-dimer [7]. Ultrasound is the initial imaging preference with a sensitivity of 80-100% and specificity of 88-98%. CT and magnetic resonance imaging are preferred if complications are suspected as they bestow further illumination regarding nearby organ infarction or thrombus extension [8]. Treatment involves anticoagulation with heparin or low-molecular-weight heparin or unfractionated heparin for three to six months based on assessment of risk factors both from COVID-19 severity and underlying medical conditions, probability of portal vein recanalization, and presence of PVT complications [3,8].

## Conclusions

Given how rarely PVT has been occurring in the setting of COVID-19 and its nonspecific presentation, it can be easily misdiagnosed or overlooked. However, given its devastating sequelae, it should be considered a differential during the investigation of gastrointestinal manifestations of the widely sweeping and highly prevalent SARS-CoV-2.
